# Study on In-Plane Compressive Buckling Behavior and Parameter Optimization of PMMA-Based Thermoplastic Sandwich Structures

**DOI:** 10.3390/ma19163525

**Published:** 2026-08-20

**Authors:** Guangtao Li, Xiaofeng Guo, Yifan Wang, Lei Zhou, Jianmin Zhang

**Affiliations:** 1 School of Intelligent Mechatronics Engineering, Zhongyuan University of Technology, Zhengzhou 450007, China; lgtdqly@163.com (G.L.); wiyifanwyf@163.com (Y.W.); 2 Longzihu New Energy Laboratory, Zhengzhou Institute of Emerging Industrial Technology, Zhengzhou 450000, China; leizhou@ipezz.ac.cn; 3 Beijing Key Laboratory of Ionic Liquids Clean Process, Institute of Process Engineering, Chinese Academy of Sciences, Beijing 100190, China

**Keywords:** wind turbine blade, polymethyl methacrylate (PMMA), sandwich web, in-plane compressive buckling, groove shape, multi-objective optimization

## Abstract

Thermosetting epoxy resins commonly used in wind turbine blades pose significant recycling challenges. This study addresses this limitation by using an eco-friendly, recyclable liquid polymethyl methacrylate (PMMA) resin to fabricate thermoplastic sandwich panels and by investigating their in-plane compressive buckling behavior. The experimental results demonstrated that the proposed PMMA thermoplastic sandwich panels exhibited improved in-plane compressive performance, with a 5.22% higher ultimate load than traditional epoxy counterparts. Furthermore, to investigate the effect of groove configuration on the buckling stability of composite sandwich panels, a finite element (FE) model for PMMA sandwich panels with initial geometric imperfections was established in this paper, and the reliability of the FE model was validated via compression and buckling tests. Finally, a Kriging surrogate model coupled with the NSGA-II algorithm was adopted to carry out multi-objective optimization, with groove parameters set as design variables. Based on the FE verification results, the optimized configuration (Point A) reduced the structural mass by 2.24%, while increasing the critical buckling load and shear modulus by 5.71% and 10.27%, respectively. Research on the buckling performance and groove configurations of PMMA sandwich panels, which can be applied to wind turbine blade webs and airfoils, can provide crucial data support for the engineering application of sustainable PMMA-based large-scale wind turbine blades.

## 1. Introduction

Wind energy represents a highly promising renewable resource characterized by its inexhaustible characteristic. At present, the vast majority of wind turbine blades are fabricated from epoxy resin matrix composites [1,2]. However, the irreversible three-dimensional cross-linked covalent networks established by post-curing render these thermosetting epoxies incapable of undergoing secondary thermal processing or melt-molding, whilst exhibiting extreme resistance to degradation in natural environments [3]. Consequently, the decommissioning of these massive epoxy-based blades generates substantial volumes of solid waste, thereby posing a severe environmental challenge. It is estimated that, by 2050, the cumulative total amount of decommissioned wind turbine blades will exceed 43 million tons, which will severely restrict the sustainable development of new energy [4].

Compared with epoxy resin composites, thermoplastic matrix composites feature characteristics such as reprocessability, room-temperature curability [5], short curing time [6], and recyclability [7,8,9], and are regarded as emerging materials for sustainable development [10,11]. In recent years, Obande et al. [12] fabricated a new Elium thermoplastic composite using the vacuum infusion process; multiaxial mechanical testing confirmed that this material exhibited better transverse tensile, flexural, and interlaminar shear properties than conventional epoxy matrix composites. Pan et al. [13] prepared thermoplastic laminates and conducted impact tests, finding that the arrangement of different foam layers affected the energy absorption capacity of the structure, while an increase in the skin fiber thickness could further enhance the impact resistance. Zhang et al. [14] developed PMMA/MMA binary liquid resins and investigated the effects of PMMA molecular weight and concentration on the resin viscosity. On the PMMA-based thermoplastic composite, Liu et al. [15] evaluated the influence of BPO initiator concentration on the mechanical properties of the composites, and compared with epoxy/GF composites, the tensile strength and flexural strength of the PMMA/GF composites increased by 18.8% and 27.4%, respectively. Additionally, Guo et al. [16] studied biaxial glass fiber-reinforced PMMA thermoplastic composites, with the results indicating that the overall mechanical properties of the PMMA composite laminates were higher than those of conventional epoxy matrix composites. To further expand the application of this material in structural components, Wang et al. [17] optimized the VARI resin infusion and curing process parameters using the response surface methodology (RSM). The optimized PMMA thermoplastic sandwich panels showed higher flexural strength, flatwise compressive strength, and edgewise compressive strength than the epoxy sandwich panels. In terms of recyclability, Gao et al. [18] established an MMA solvent recovery process, where separation of the resin and fibers in GF/PMMA composites was achieved after immersion at 60 °C for 32 h. The recovered glass fibers can be directly reused, enabling a closed-loop recycling pathway. Ren et al. [19] extended this method to CFF-reinforced HGM/PMMA sandwich composites, achieving a three-phase separation of the carbon fiber fabrics, PMMA matrix, and HGM microspheres through MMA immersion at room temperature for 36 h. The retention rate of the mechanical properties for the recycled sandwich panels exceeded 96%, with a modulus degradation of less than 2%. This solvent-based recycling approach can be implemented under mild operating conditions. It circumvents fiber degradation and excessive energy consumption inherent to conventional treatment routes, thereby offering a viable strategy for the reclamation of composite materials.

These advances in material performance and recyclability further broaden the engineering potential of thermoplastic sandwich composites. Owing to their high specific stiffness and lightweight characteristics, composite sandwich structures have been employed in a wide range of engineering applications. In the aerospace field, Redmann et al. [20] investigated carbon-fiber composite sandwich panels with aluminum and aramid honeycomb cores for airframe structures, demonstrating their application in lightweight aircraft structures. In the marine field, Di Bella et al. [21] investigated glass-fiber-reinforced sandwich structures used in luxury-yacht components, demonstrating their applicability to lightweight marine structures. In civil infrastructure and equipment, FRP sandwich systems have been developed for building roofs and structural cladding, wind turbine blades, bridge decks, beams and girders, and railway components [22]. More recently, Mazzuca et al. [23] investigated the fire resistance of vacuum-infused GFRP sandwich panels with polyurethane (PUR) and polyethylene terephthalate (PET) foam cores under sustained bending loads, further demonstrating the broad application potential of these materials in the construction sector. Against this broad engineering background, the present study focuses specifically on sandwich structures used in large-scale wind turbine blade shear webs.

Modern large-scale wind turbine blades typically utilize a box-girder structural configuration, whose primary load-carrying system is composed of upper and lower spar caps together with shear webs. As thin-walled components characterized by a high span-to-thickness ratio, the webs exhibit a buckling failure load that is substantially lower than the ultimate compressive load [24]. Regarding the stability of composite sandwich structures, several full-scale and component-level experimental studies indicate that structural geometric constraints, matrix material properties, and load distributions are the primary factors influencing the in-plane compressive buckling of the webs. Mishnaevsky [25] reported that when a sandwich structure enters the post-buckling stage, local buckling instability leads to a reduction in structural stiffness. Zhang et al. [26] combined finite element simulations with destructive testing and found a coupling effect between the large deformations induced by web component instability and the global failure of the main girder. Li et al. [27] and Zhang et al. [28] conducted experimental investigations under combined loading conditions and found that the in-plane compressive load acting on the web edges is a factor determining the structural instability of the blade.

To improve the buckling resistance of sandwich structures under in-plane compressive loads, researchers have investigated various foam configuration methodologies. Mouritz [29] reviewed the reinforcement mechanisms of 3D Z-pinning technology in sandwich materials, noting that the introduction of orthogonal or inclined fiber/metal pins in the thickness direction enhances the interlaminar bonding between the skins and the foam. Cao et al. [30] explored a foam configuration approach using 3D-printed foams via fused deposition modeling (FDM), regulating resin absorption at the skin–foam interface through a compression molding process; this approach led to structural mass reduction while increasing the out-of-plane and in-plane shear strengths by 25% and 34%, respectively. Zhang et al. [31] investigated the effects of geometric configuration optimization of truss foams on the performance of the overall sandwich structure. Balıkoğlu et al. [32] experimentally found that both perforated and grooved foams enhance the flexural strength and effective stiffness of sandwich beams, with the perforation process showing a more pronounced increase in the ultimate flexural strength. Furthermore, Qin et al. [33,34,35] systematically examined the influences of different foam processing configurations on the mechanical properties of epoxy sandwich panels, demonstrating that prefabricating grooves of specific geometries within the foam and filling them with resin increases the compressive and shear moduli of the foam.

Literature reviews indicate that current research on PMMA thermoplastic sandwich composites is primarily confined to resin formulation modification and strength characterization. Given that the buckling instability of sandwich panels is a prevalent and catastrophic failure mode, investigating the buckling stability characteristics of PMMA sandwich panels is paramount to promote the scalable application of recyclable PMMA thermoplastic resins in wind turbine blades. Additionally, current studies on the effects of foam configuration parameters on the buckling stability of sandwich panels mostly utilize single-factor analysis methods. However, these configuration parameters exert complex interactive effects on both the structural stability and the structural mass. Consequently, it is of great significance to optimize the foam configuration parameters of sandwich panels through multi-parameter and multi-objective optimization methodologies based on surrogate models.

Based on the aforementioned issues, this study adopts the shear web of large-scale wind turbine blades as the engineering prototype to design and fabricate PMMA/GF/PET sandwich panel components. A conventional epoxy thermosetting sandwich panel is introduced as a reference, and in-plane compressive buckling tests are systematically conducted. First, through quasi-static compression experiments, the response differences between the new PMMA thermoplastic and the conventional epoxy thermosetting sandwich web regarding the buckling initiation load, ultimate load, and post-buckling load-carrying characteristics are studied. Second, a geometrically nonlinear FE model incorporating initial geometric imperfections was established to predict the Critical buckling load and deformation evolution of the sandwich panels. Finally, the foam groove width, depth, and spacing were selected as design variables, while the Critical buckling load, effective shear modulus, and structural mass were adopted as the optimization responses.

## 2. Experimental Section

### 2.1. Experimental Materials

The materials requisite for thermoplastic resin fabrication encompass: polymethyl methacrylate (PMMA) exhibiting a number-average molecular weight (*M*n) of 1 × 10^5^ g/mol, purchased from Dongguan Hongxing New Materials Co., Ltd. (Dongguan, China); methyl methacrylate (MMA; 99.0% purity), the initiator benzoyl peroxide (BPO; 99.0% purity) and N,N-dimethylaniline (DMA), all of which were obtained from Shanghai Macklin Biochemical Technology Co., Ltd. (Shanghai, China); 190/195 epoxy resin, acquired from Shanghai Daosheng Tianhe Materials Technology Co., Ltd. (Shanghai, China); T80 PET foam, supplied by Beijing Zhongnuo Composite Materials Co., Ltd. (Beijing, China); and biaxial fiber fabric EKB800(+45°/−45°)TP-1260 possessing an areal density of 800 g/m^2^, sourced from Chongqing International Co., Ltd. (Chongqing, China).

### 2.2. Preparation of Test Specimens

#### 2.2.1. Resin Structural Components

The binary liquid mixed resin was prepared at a PMMA:MMA mass ratio of 24:100. The reducing agent DMA and the oxidizing agent BPO were sequentially added according to an MMA:BPO:DMA molar ratio of 200:1.2:1, and the mixture was stirred slowly to ensure complete dissolution of the initiator components. For the preparation of the epoxy resin, epoxy resin 190 and curing agent 195 were mixed in a beaker at a mass ratio of 100:28. The mixture was thoroughly stirred and then placed in a vacuum oven for 5 min to eliminate air bubbles. Subsequently, the PMMA resin and epoxy resin were poured into molds. Specifically, the PMMA resin sheet was cured in an oven at 35 °C for 12 h and subsequently at 80 °C for 2 h, while the epoxy resin was cast into a mold and solidified at 60 °C for 8 h, followed by 100 °C for 2 h. The mechanical properties of the PMMA resin and epoxy resin are presented in Table 1 [15]. The material properties of T80 PET foam used in the finite element simulations are listed in Table 2.

#### 2.2.2. Laminated Structural Components

To meet the requirements of mechanical property tests, PMMA and Epoxy laminates were fabricated. All infusions were conducted at room temperature, followed by a two-step curing process. For the PMMA/GF components [18], the laminates were cured at 35 °C for 8 h and then at 80 °C for 4 h. For the Epoxy/GF components, the curing process was carried out at 50 °C for 4 h and then at 70 °C for 10 h.

The equivalent elastic parameters of the unidirectional PMMA/GF and Epoxy/GF laminates are presented in Table 3. Among these, *E*_1_, *E*_2_, and *v*_12_ were acquired through the tensile tests in 0° and 90°directions, respectively, and the test methods adhere to ASTM D3039 [36]; *G*_12_ was obtained via the in-plane shear test, and the test methods follow ASTM D3518 [37]. Given that the finite element analysis in this paper primarily focuses on the overall elastic buckling response and critical buckling load prediction of the sandwich web, the parameters related to the thickness direction are determined based on the transverse isotropy assumption of the unidirectional laminate and the conversion of engineering constants. Therefore, *E*_2_ = *E*_3_, *v*_12_ = *v*_13_, *G*_12_ = *G*_13_, and *G*_23_ = *E*_2_/[2(1 + *v*_23_)].

#### 2.2.3. Structural Components of Sandwich Panels

**Figure 1:**
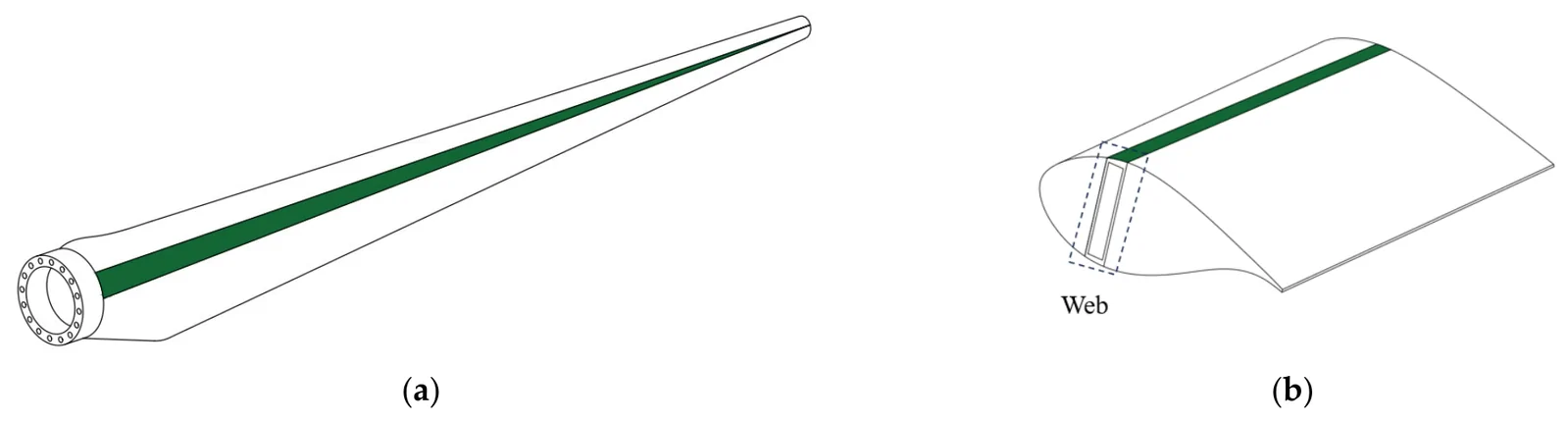
(**a**) Full-scale blade. (**b**) Blade segment.

Modern large-scale wind turbine blades typically utilize a box-girder load-bearing structure, which is mainly composed of upper and lower spar caps and shear webs, as illustrated in Figure 1. Within this framework, the spar caps primarily bear the tensile and compressive stresses induced by the blade bending loads, whereas the shear webs function to transfer shear loads, maintain cross-sectional stability, and cooperatively constrain the upper and lower spar caps. Due to the high span-to-thickness ratio of the web sandwich structures, failure modes such as global buckling, local buckling, and foam crushing are prone to occur under in-plane compressive loading. Therefore, this study extracts the typical sandwich load-bearing unit from the shear web to conduct in-plane compressive buckling research.

**Figure 2:**
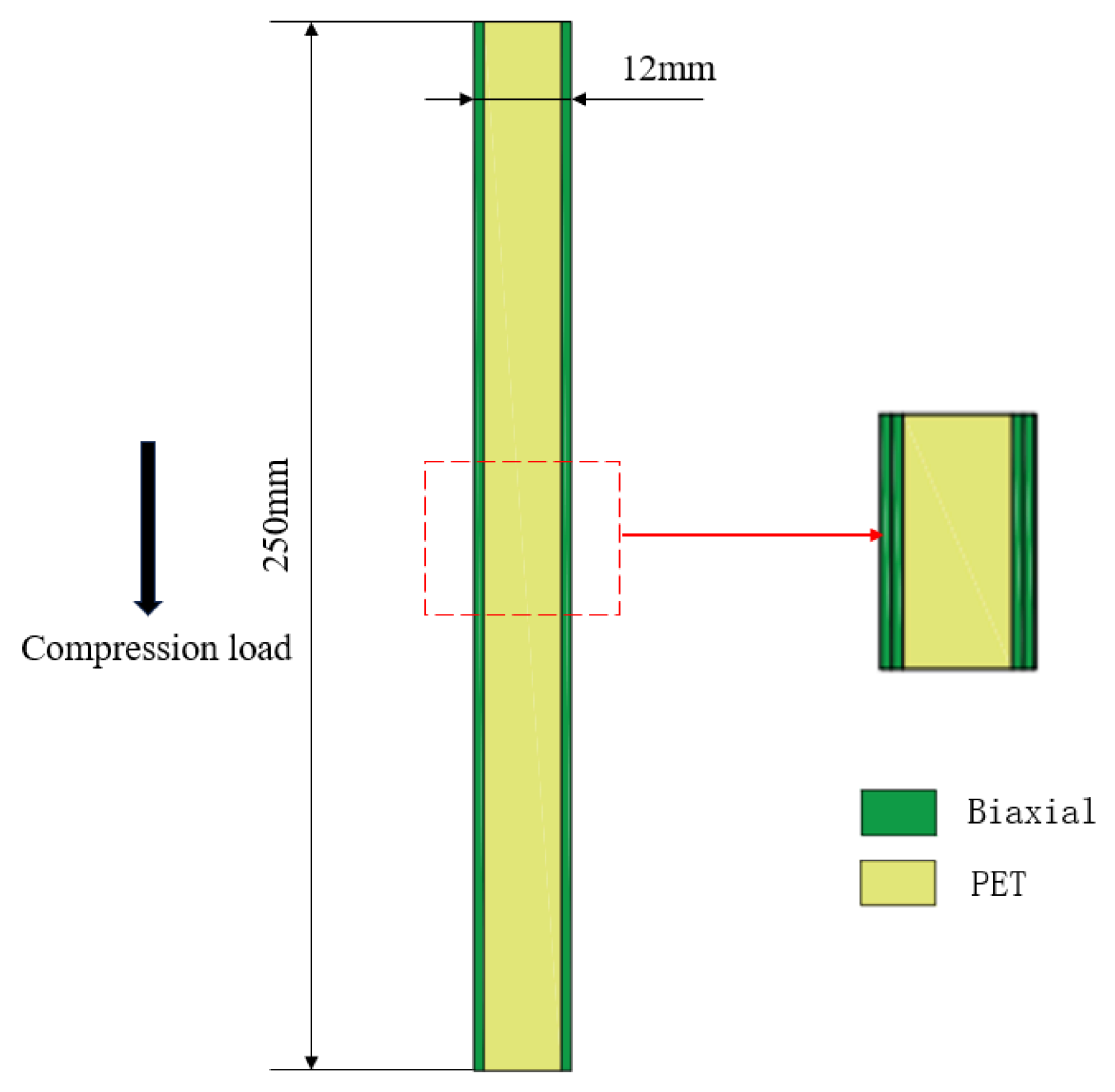
Schematic diagram of a web component.

Figure 2 presents the schematic diagram of the sandwich panel designed in this study. Both the PMMA sandwich composite panel (PMMA/SCP) and epoxy sandwich composite panel (Epoxy/SCP) specimens adopted a GF/PET/GF sandwich configuration. The compression specimens were 250 mm in height, 100 mm in width, and 12 mm in total thickness, with the height direction defined as the loading direction. Each face sheet consisted of two plies of EKB800 biaxial glass-fiber fabric with +45°/−45° fiber orientations relative to the loading direction. The nominal cured thickness of each biaxial ply was 0.5 mm, giving a total face-sheet thickness of 1.0 mm. The T80 PET foam core was 10 mm thick, resulting in a through-thickness configuration of 1.0 mm/10 mm/1.0 mm for the front face sheet, foam core, and rear face sheet, respectively.

**Figure 3:**
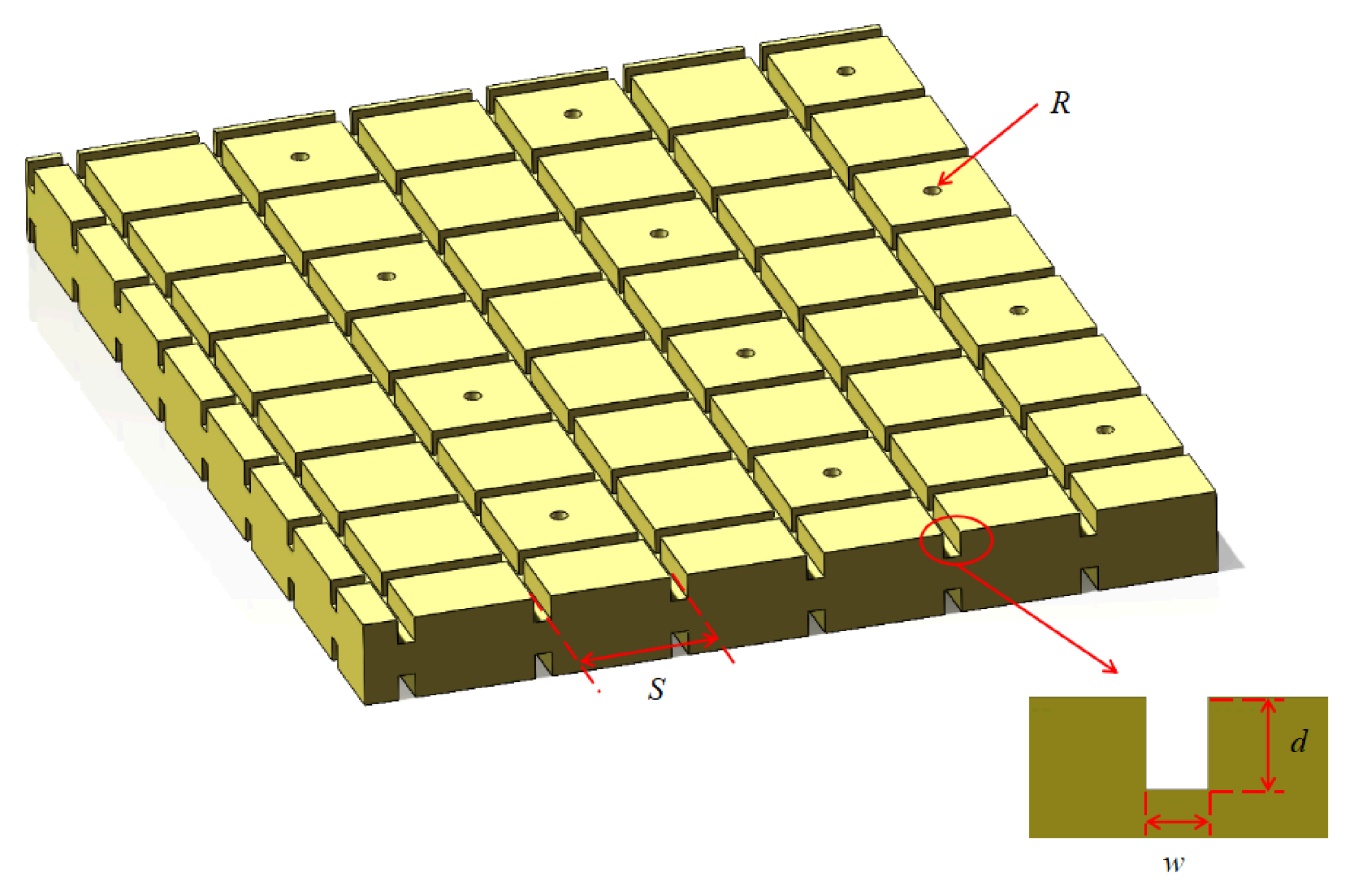
Processing Configuration of the foam.

The T80 PET foam used in the baseline PMMA/SCP and Epoxy/SCP specimens was machined with orthogonal grooves and circular perforations, as illustrated in Figure 3. The groove spacing was 16 mm in both in-plane directions *(s* = 16 × 16 mm), the groove width was *w* = 2 mm, the groove depth was *d* = 3 mm, and the perforation radius was *R* = 1 mm. This groove–perforation configuration was adopted as the baseline configuration for both the experiments and the corresponding FE model, and its geometric parameters are summarized in Table 4.

**Figure 4:**
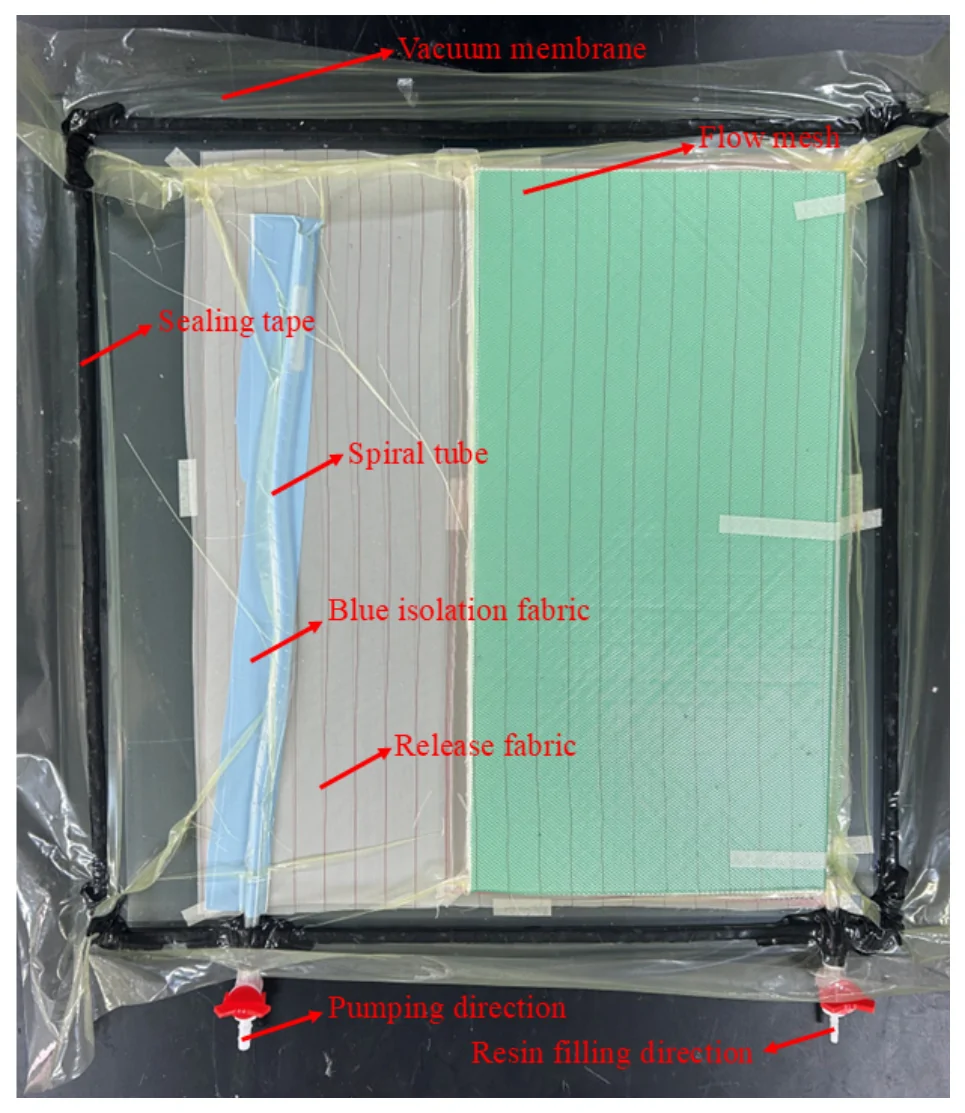
Vacuum Infusion Process.

Both types of sandwich panels were fabricated using the vacuum infusion process [17]. The vacuum infusion setup and resin flow arrangement used for panel fabrication are shown in Figure 4. A two-stage curing process was applied to the PMMA thermoplastic resin system: the panels were first cured at 30 °C for 8 h, followed by post-curing at 80 °C for 2 h, ensuring sufficient polymerization of the PMMA/MMA resin and improving the composite molding quality. The epoxy resin system similarly utilized a two-stage curing process, consisting of initial curing at 60 °C for 8 h followed by post-curing at 100 °C for 2 h.

### 2.3. Numerical Model

**Figure 5:**
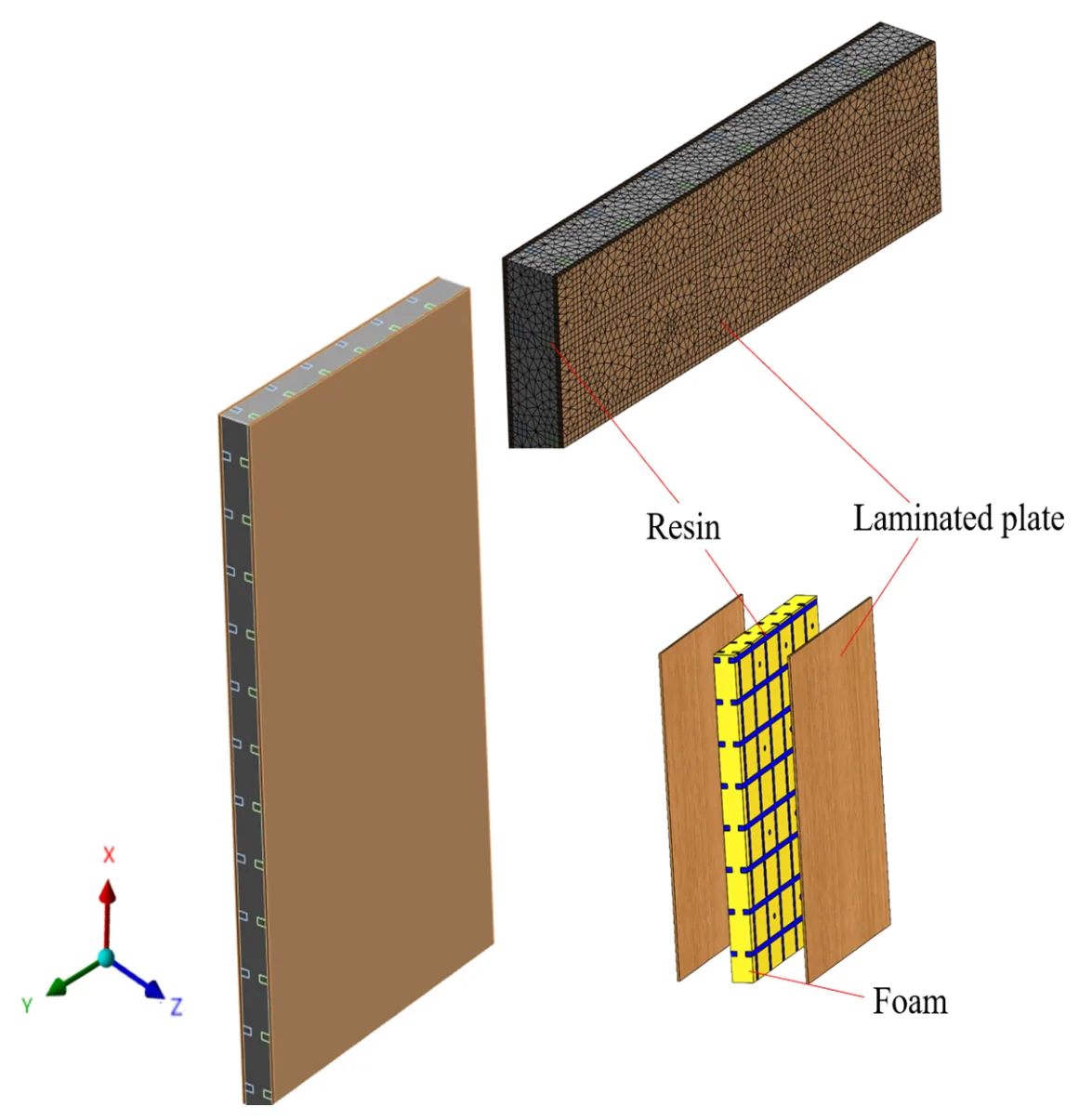
Finite Element Model.

In this paper, during the modeling process, a layer of biaxial fiber is considered equivalent to two unidirectional (UD) sub-layers, specifically [+45°/−45°/+45°/−45°/PET]_s_ [38]. According to the prescribed core-machining configuration, the grooved and perforated core geometry was first constructed in SOLIDWORKS 2021 and subsequently imported into ANSYS 2024R2. The grooves and perforations were assumed to be completely filled with PMMA resin. The remaining PET foam and the PMMA resin occupying the grooves and perforations were represented as separate solid bodies, while the upper and lower composite face sheets were generated independently using the ACP module. The corresponding material properties were assigned to the laminate, PET foam, and resin-filled regions, respectively. Since no interfacial debonding was observed during the experiments, the adjacent components were assumed to be perfectly bonded and were connected using bonded constraints. The finite element model and mesh division of the sandwich panel are presented in Figure 5.

All numerical simulations were performed using the implicit static solver in ANSYS Mechanical APDL, with the symmetric sparse direct solver adopted for matrix solution. The SOLID186 20-node hexahedral solid elements with uniform reduced integration (KEYOPT(2) = 0) were employed to alleviate shear locking and improve the accuracy of bending and buckling analyses.

### 2.4. Boundary Conditions

In the buckling analysis, a two-stage procedure was adopted to investigate the buckling response of the sandwich panels. To approximate the end restraint and load-transfer conditions produced by the rigid compression platens after specimen seating, the lower end face was fully constrained, while the upper end face was rigidly coupled to a remote point located at its geometric center through multipoint-constraint equations.

In the first stage, a prestressed static analysis followed by a linear eigenvalue buckling analysis was performed to determine the critical instability modes. At the upper remote point, the transverse translations and all rotational degrees of freedom were constrained: *U*_Y_ = *U*_Z_ = *θ*_X_ = *θ*_Y_ = *θ*_Z_ = 0, while the axial translation *U*_X_ remained free. A reference compressive force of *F*_X_ = −1 N was applied along the axial direction. Since the magnitude of the reference compressive force was 1 N, the first positive buckling load factor was numerically equal to the first-order eigenvalue buckling load in Newtons.

In the second stage, the imperfect geometry was transferred to a geometrically nonlinear static analysis with large-deformation effects considered. The lower-end constraint, the rigid remote-point coupling, and the transverse and rotational constraints at the upper remote point were retained: *U*_Y_ = *U*_Z_ = *θ*_X_ = *θ*_Y_ = *θ*_Z_ = 0. The reference compressive force used in the eigenvalue analysis was removed and replaced by a prescribed axial displacement of *U*_X_ = −6 mm.

### 2.5. Mesh-Convergence Verification

A mesh-convergence study was conducted using seven nominal mesh sizes of 5.0, 4.0, 3.0, 2.0, 1.0, 0.8, and 0.7 mm. As summarized in Table 5, the predicted Critical Buckling Load gradually converged as the mesh was refined. Taking the result obtained with the finest 0.7 mm mesh as the reference, the relative error decreased from 32.60% for the 5.0 mm mesh to only 0.55% for the 1.0 mm mesh. Further refinement from 1.0 mm to 0.7 mm resulted in only a marginal change in the predicted critical buckling load, from 18.40 to 18.30 kN. Therefore, a nominal mesh size of 1.0 mm was adopted for the subsequent buckling analyses as a compromise between numerical accuracy and computational efficiency.

In the ACP model, each 0.5 mm-thick biaxial glass-fiber fabric ply was represented by two equivalent 0.25 mm thick unidirectional analysis plies oriented at +45° and −45°, respectively. The Analysis Ply Wise extrusion method was adopted, such that each analysis ply was extruded into one solid-element layer. Consequently, each 1.0 mm thick composite face sheet contained four solid-element layers through its thickness. The 10 mm thick foam core contained ten elements through its thickness. The final finite element model comprised 480,257 elements.

### 2.6. Experimental Setup

**Figure 6:**
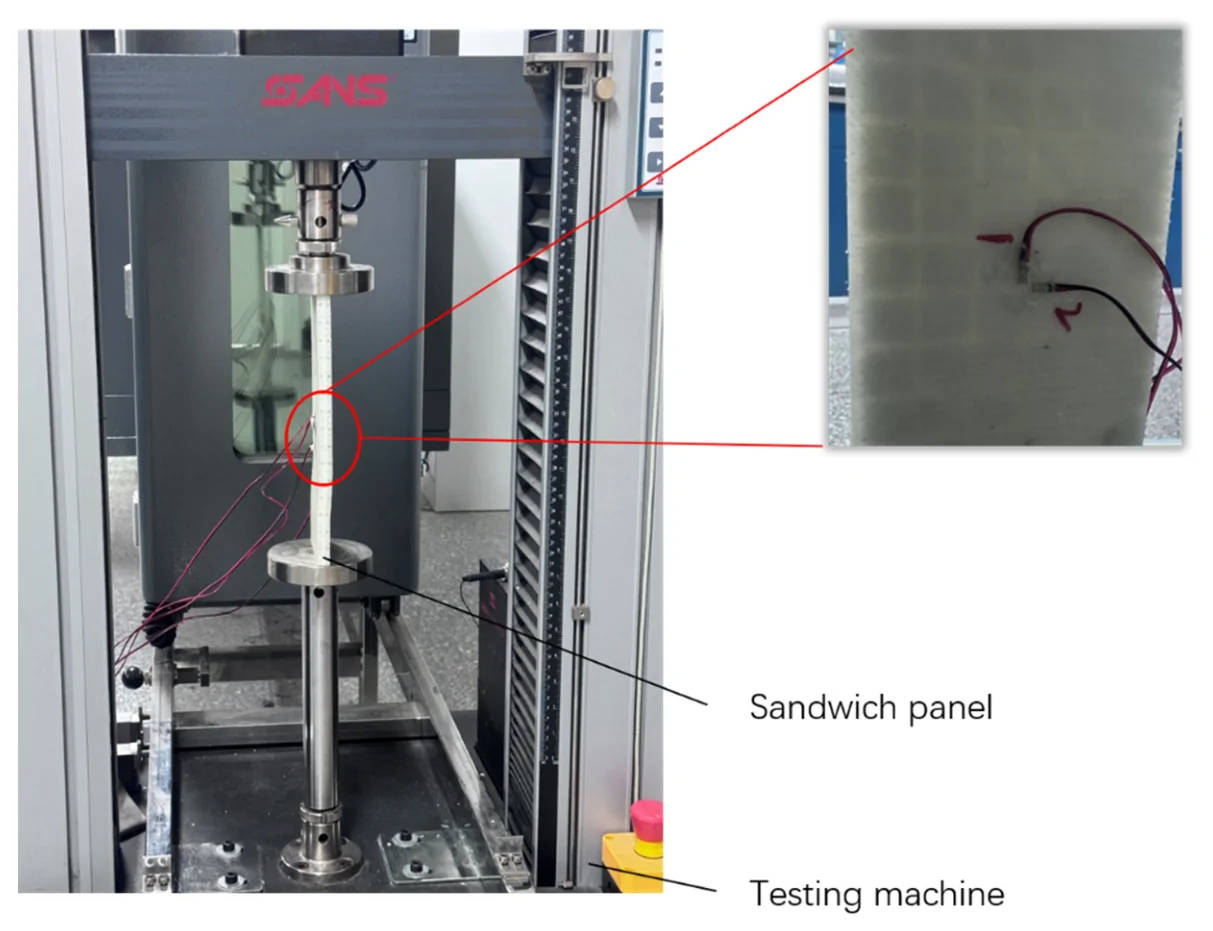
Experimental setup for buckling tests on sandwich panels.

In this study, a general compression test platform was constructed based on a microcomputer-controlled electronic universal testing machine with a maximum design load of 30 kN. The experimental system is presented in Figure 6. The test was conducted under the displacement control mode for quasi-static loading. The hydraulic actuator moved at a constant rate of 1 mm/min to guarantee that the response characteristics after structural instability could be stably monitored and the influence of the strain-rate effect on the mechanical properties of the material could be eliminated.

To document the structural response characteristics of the specimen during the experiment, uniaxial foil strain gauges (Model: BX120-3AA, resistive grid size: 3 mm × 2 mm, nominal resistance: 120 Ω, RunesKee, Shenzhen, China) were affixed at the centers of the two panels of the test piece, configured in a half-bridge circuit with identical compensation gauges to eliminate temperature-induced apparent strain. These strain gauges were used to collect strain data in real time in two directions, aligned with the compression direction (designated as direction 1, longitudinal) and perpendicular to it (designated as direction 2, transverse). A high-speed strain testing and analysis system with 4 acquisition channels was used for strain data acquisition. The maximum continuous sampling rate was 1 kHz, which met the requirements for capturing rapid strain changes during the instability process. Given that the instability process was relatively brief, a camera was positioned directly in front of the specimen to visually capture the change process of the sandwich panel specimen in real time.

## 3. Results and Discussion

For clarity, the characteristic loads used in the following discussion are defined first. The buckling initiation load is defined as the load corresponding to the first sustained deviation of the pre-peak load–displacement response from the approximately linear trend, at which the slope of the curve, namely the structural tangent stiffness, begins to decrease continuously [39,40]. The ultimate load is defined as the maximum load recorded on the experimental load–displacement curve. Beyond this point, the specimen displacement continues to increase without a further increase in load, followed by a reduction in load-carrying capacity and global buckling failure. In the numerical simulations, the corresponding numerical buckling load is termed the critical buckling load to distinguish it from the experimentally determined characteristic loads. Based on these definitions, the compressive buckling evolution and failure modes of the specimens are discussed below.

### 3.1. Compressive Buckling Failure Evolution and Failure Modes

**Figure 7:**
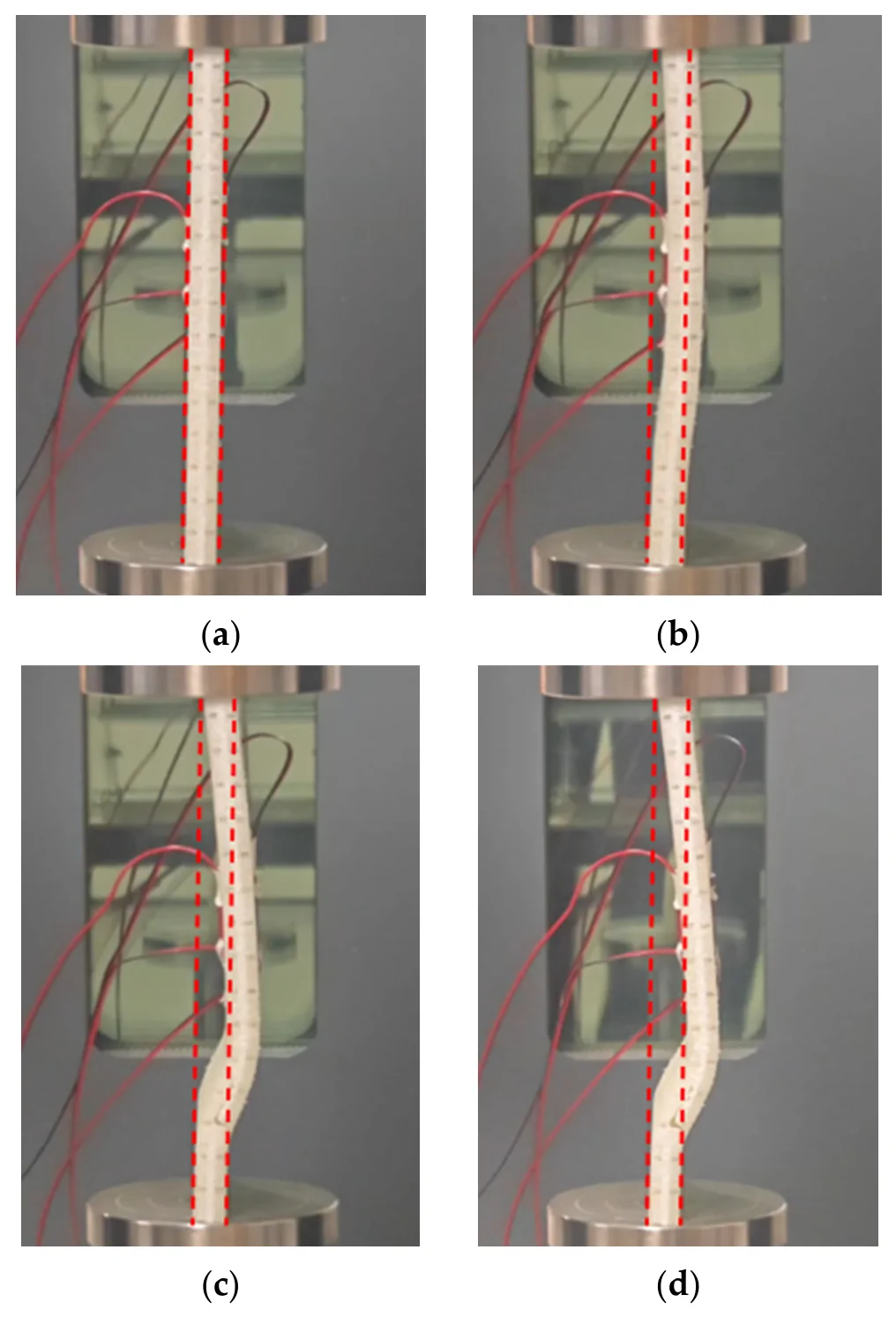
Buckling failure process of the sandwich panels under axial compression: (**a**) initial loading stage, with no obvious lateral deflection; (**b**) onset of buckling deformation; (**c**) progressive development of out-of-plane deflection during compression; (**d**) final buckling failure state with pronounced lateral deflection. The red dashed lines indicate the reference lines used to visualize the lateral deformation during buckling.

To investigate the buckling behavior of the PMMA/SCP and Epoxy/SCP sandwich panels under axial compressive loading, data encompassing load–displacement curves and specimen failure modes were acquired utilizing the aforementioned experimental apparatus. Combined with empirical observations, the mechanical response characteristics of both sandwich configurations were scrutinized, followed by a statistical analysis of the experimental datasets. Under axial compression, both types of sandwich panels began to exhibit buckling deformation after reaching their respective buckling initiation loads. With further loading, the buckling deformation developed progressively until the specimens reached their ultimate loads and eventually underwent global buckling failure. The representative buckling failure sequence of the PMMA sandwich panels is illustrated in Figure 7.

Under compressive loading, all specimens began to exhibit buckling deflections after reaching their respective buckling initiation loads. To further scrutinize the post-buckling failure modes of the PMMA sandwich panels, loading was continued beyond the ultimate load until global buckling failure occurred. The progressive failure sequence was characterized as follows: during the initial loading phase, the load escalated rapidly without any detectable lateral displacement, signifying a linear elastic regime. As the force reached approximately 51.41% of the peak load, the slope of the load–deflection curve gradually attenuated, accompanied by the onset of minor lateral deflections within the sandwich configurations. Upon further loading to roughly 93.66% of the ultimate load, the load increment rate decelerated profoundly, and conspicuous macro-buckling deformations became visually prominent. Following the attainment of the peak load, the lateral displacement amplified abruptly with an accelerating pace, precipitating a gradual loss in structural stability. Concurrently, localized crushing and fracturing within the internal foam were observed, which immediately triggered the catastrophic failure of the sub-components. At the final stage of global buckling failure, the panel manifested severe macroscopic bending; nevertheless, the outer composite skin remained completely intact without any visible microcracks or delamination. This failure topology, predominantly concentrated in the cracking and crushing of the mid-span foam, substantiates that the ultimate load-carrying capability of the web components is decisively dictated by the structural integrity of the internal foam matrix. Consequently, the definitive failure mechanism of these sandwich configurations is categorized as global buckling collapse. The comprehensive dataset extracted from the compressive buckling evaluations is summarized in Table 6.

As indicated in Table 6, all specimens exhibited global buckling failure. The average buckling initiation load and ultimate load of the PMMA/SCP sandwich panels were 9.06 kN and 17.93 kN, respectively, whereas the corresponding indices for the Epoxy/SCP sandwich panels were 7.42 kN and 17.04 kN.

### 3.2. Load–Displacement Response and Numerical Model Validation

(1) The load–displacement responses

**Figure 8:**
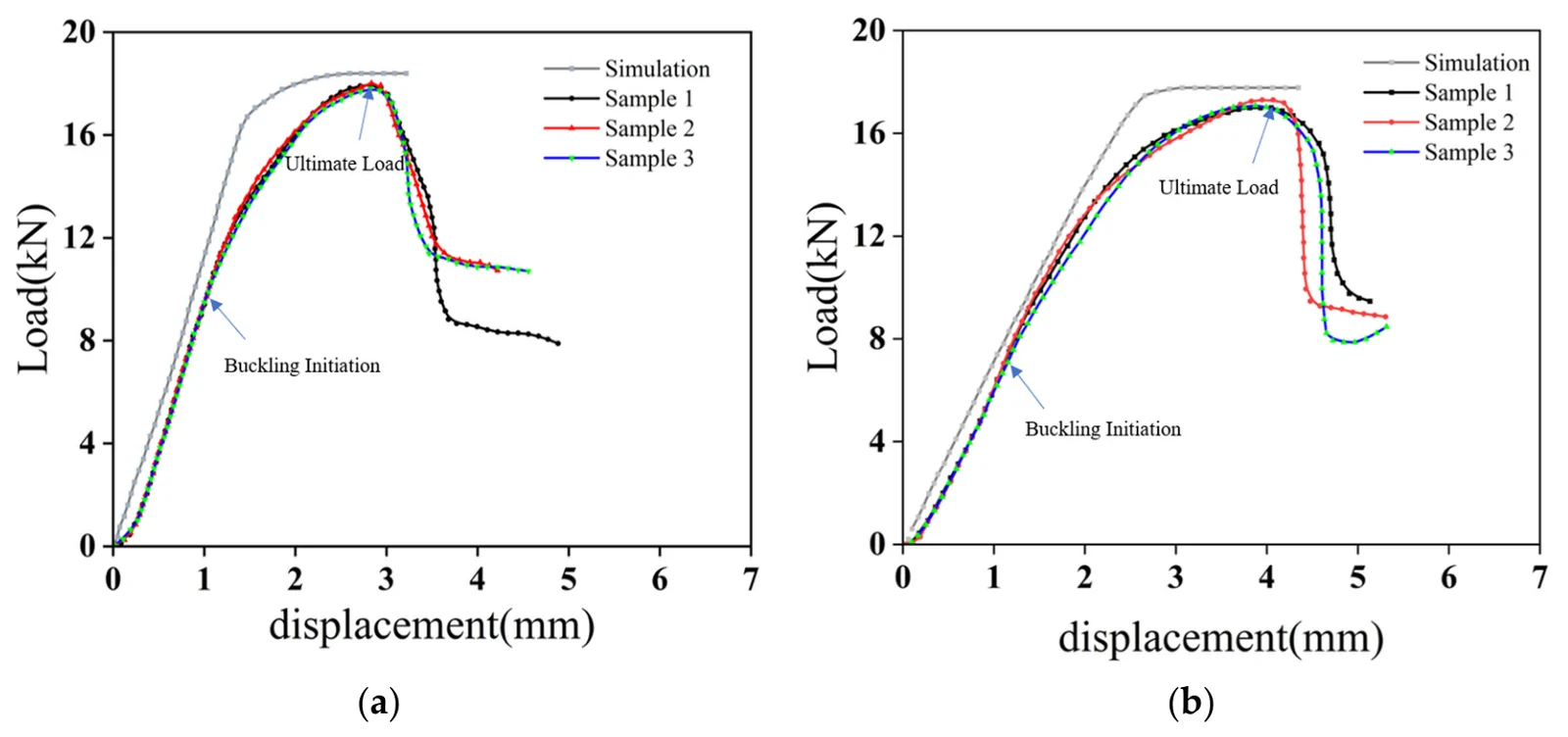
Comparison of in-plane compressive load–displacement curves between experimental testing and finite element (FE) simulation: (**a**) PMMA/SCP sandwich panels; (**b**) Epoxy/SCP sandwich panels.

The load–displacement responses of the PMMA/SCP and Epoxy/SCP sandwich panels captured via the cross-head displacement sensor under axial compressive loading are depicted in Figure 8. As illustrated in Figure 8a,b, the mechanical profiles of both sandwich configurations can be systematically partitioned into three distinctive stages: the initial nonlinear region, the pre-peak linear elastic regime, and the post-peak load-carrying degradation zone. In the initial nonlinear stage, the load increased nonlinearly with displacement, mainly due to the initial seating/contact between the specimen ends and the compression platens. After stable contact was established, the specimens entered the pre-peak linear elastic stage, in which the load increased approximately linearly with displacement. After the ultimate load was reached, the load decreased with increasing displacement as buckling deformation developed and the core underwent local crushing, indicating a progressive loss in load-carrying capacity.

Correlating the comparisons from Table 6 and the response trajectories in Figure 8, it can be discerned that the PMMA thermoplastic sandwich structures exhibited a pronounced superiority in their in-plane compressive mechanical performance. Specifically, their mean buckling initiation load and average ultimate load increased by 22.10% and 5.22%, respectively, relative to the Epoxy/SCP counterparts. This phenomenon is intrinsically ascribed to the highly cross-linked network morphology of the epoxy matrix; such a dense cross-linking characteristic inadvertently restricts molecular chain movement, thereby leading to insufficient energy dissipation capacity under load and rendering the specimens susceptible to localized stress concentrations and premature failure within the contact zones. In sharp contrast, the PMMA thermoplastic resin possesses a linear macromolecular chain architecture, which is highly conducive to intermolecular energy transfer. When the PMMA/SCP composites undergo loading, stress can be effectively dissipated from the loading zone to the peripheral regions, thereby expanding the effective load-bearing area and prolonging the duration of load sustenance [16]. Furthermore, during the post-peak stage upon traversing the ultimate load, the Epoxy/SCP structures experienced a sharp drop in load with a compromised residual bearing capacity, which represents a typical consequence of brittle fracture propagation within thermosetting matrices. Conversely, the load attenuation curve of the PMMA/SCP configurations was far more gradual, demonstrating superior post-buckling load-carrying stability and energy absorption potential.

(2) Imperfection-amplitude sensitivity analysis

To evaluate the influence of the assumed initial imperfection amplitude on the predicted buckling response, a sensitivity analysis was conducted using four imperfection amplitudes: 0.05% t, 0.10% t, 0.20% t, and 0.50% t, where t = 12 mm denotes the total thickness of the sandwich panel [41]. All other material properties, mesh settings, boundary conditions, and loading conditions were kept unchanged.

The corresponding predicted critical buckling loads were 19.06, 18.40, 17.25, and 14.56 kN, respectively. The results show that the predicted Buckling Load decreases with increasing imperfection amplitude, indicating that the sandwich panel is sensitive to the assumed initial geometric imperfection. For the 0.10% t case adopted in the present study, the predicted Buckling Load was 18.40 kN, which differed by only 2.62% from the experimental average value of 17.93 kN (as shown in Figure 8a). Therefore, the 0.10% t imperfection amplitude was taken as the baseline value for the subsequent analyses.

(3) Numerical Model Validation

As illustrated in Figure 8a,b, the numerical curves primarily reproduce the pre-peak response. Because the numerical boundary conditions idealize the platen–specimen contact, the initial seating/contact nonlinearity observed experimentally is not explicitly represented, and the post-peak descending branch is not reproduced.

The Critical Buckling Loads of the PMMA/SCP and Epoxy/SCP specimens were 18.40 kN and 17.77 kN, respectively, corresponding to relative differences of 2.62% and 4.28% from the experimental average values. These results indicate that the developed finite element model can reasonably predict the elastic and geometrically nonlinear buckling responses of the PMMA/SCP and Epoxy/SCP configurations investigated in this study. However, because material nonlinearity, progressive damage, foam crushing failure, and interfacial damage were not incorporated into the current model, the post-peak descending branch of the experimental load–displacement response could not be reproduced. Therefore, the present validation is limited to the buckling-load prediction and deformation response of the tested configurations.

### 3.3. Displacement–Strain Response Analysis

**Figure 9:**
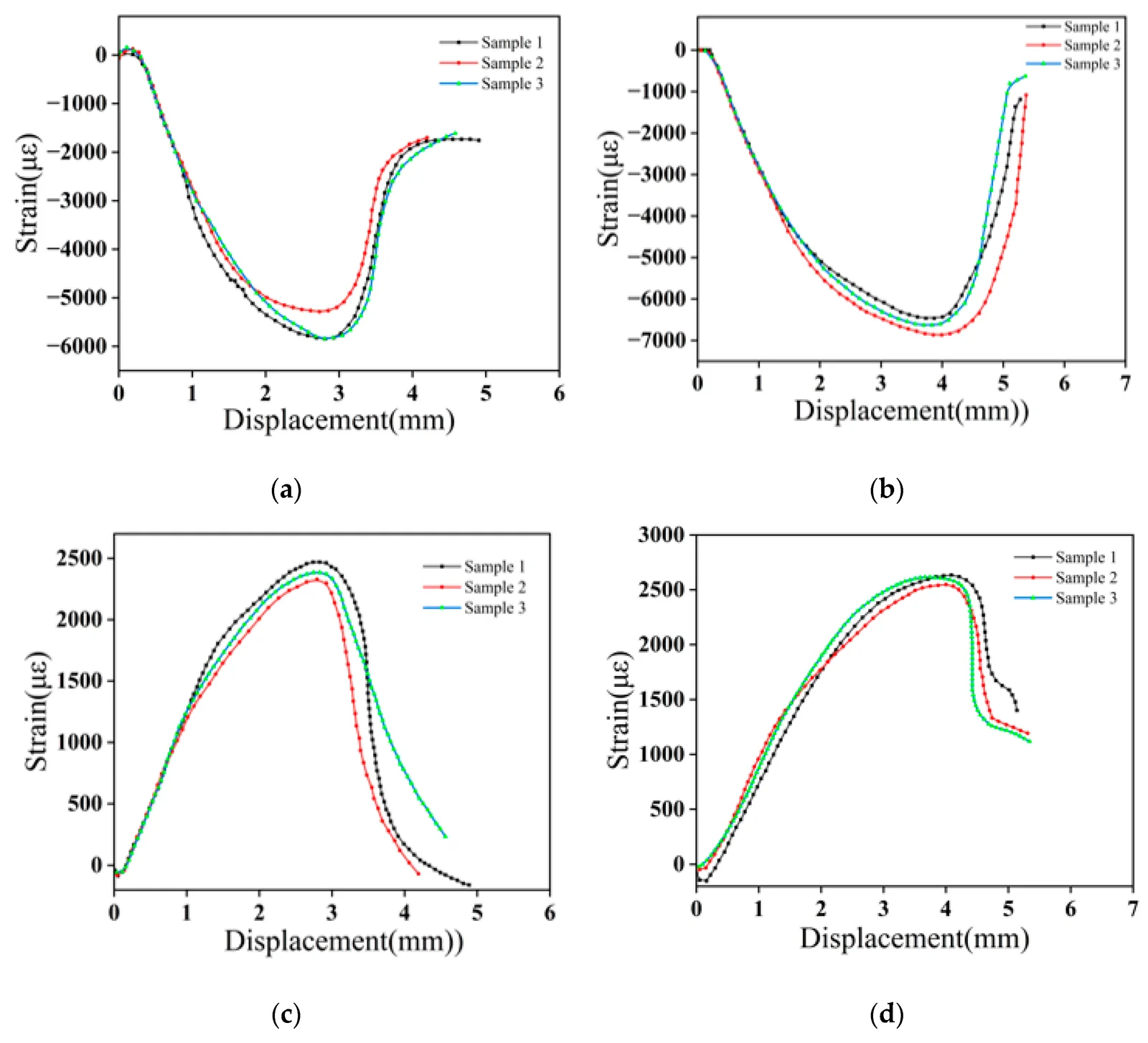
Comparison of displacement–strain curves between PMMA/SCP and Epoxy/SCP sandwich panels: (**a**) PMMA/SCP in Direction 1; (**b**) Epoxy/SCP in Direction 1; (**c**) PMMA/SCP in Direction 2; (**d**) Epoxy/SCP in Direction 2.

To further unveil the mechanical behavior and buckling failure mechanisms of the PMMA/SCP and Epoxy/SCP sandwich panels during axial compression, the axial (1-direction) and transverse (2-direction) displacement–strain response curves at the critical measuring points of the face sheets were extracted, as depicted in Figure 9.

As illustrated in Figure 9a,b, during the initial stage of axial (1-direction) compression (the linear phase), the strains of the face sheets for both sandwich configurations increased linearly with the applied displacement. When the applied load approached the respective buckling initiation loads (approximately 9.06 kN for the PMMA/SCP and 7.42 kN for the Epoxy/SCP, as summarized in Table 6), a distinct slope change occurred in both strain curves, signifying the initiation of face-sheet stiffness degradation. Comparative analysis reveals that the PMMA/SCP sandwich panels manifested a lower ultimate strain amplitude at the peak-load displacement, alongside a more gradual strain accumulation rate throughout the post-buckling stage. This phenomenon demonstrates that the superior toughness and stiffness of the PMMA matrix inherently reinforced the buckling resistance of the face sheets, thereby retarding the progressive evolution of global structural instability.

Figure 9c,d display the transverse (2-direction) displacement–strain curves. Influenced by the Poisson’s ratio effect and the complex stress state induced by compressive loading, the transverse strains of both specimen cohorts exhibited a stable, near-linear growth profile during the initial nonlinear region and the pre-peak linear elastic regime. Upon transitioning into the post-peak load-carrying degradation zone, the transverse tensile strains amplified rapidly due to the global flexural deformation of the structure, clearly reflecting the localized bulging behavior of the face sheets. Notably, the PMMA/SCP sandwich panels maintained a lower ultimate transverse strain amplitude at the peak-load displacement compared to the Epoxy/SCP references, further confirming that the superior stiffness of the PMMA matrix provides enhanced structural constraints. This superiority effectively mitigates localized face-sheet deflections and suppresses lateral expansion. Furthermore, the PMMA/SCP configurations exhibited a more stable and continuous strain transition after reaching peak displacement, which highly correlates with the macroscopic failure morphology (intact face sheets and crushed foam) observed in Section 3.1.

## 4. Multi-Objective Optimization of Foam Processing Configurations

### 4.1. Definition of the Multi-Objective Optimization Problem

The grooved configuration of the foam material not only dictates the infusion percolation and wet-out states of the PMMA resin within the PET foam but also further modifies the localized stiffness distribution, shear deformation resistance, and global instability behavior of the sandwich architectures. Consequently, appropriately configuring the geometric parameters of the foam grooves constitutes the linchpin for achieving a synergistic design that simultaneously yields high load-carrying capacity, outstanding rigidity, and light-weighting efficiency.

Focusing on the grooved PMMA/PET composite-foam sandwich panels, the present study designates the foam groove width (*w*), groove depth (*d*), and groove spacing (*s*) as continuous design variables, while selecting the critical buckling load (*P*_cr_), shear modulus (*G*_c_), and structural mass (*m*) as the optimization responses. For each groove configuration, the critical buckling load *P*_cr_ and structural mass *m* were obtained from the parameterized compression-buckling finite element model of the representative sandwich-web specimen. The effective core shear modulus *G*_c_ was evaluated using a separate shear finite element model established according to the specimen configuration and loading principle specified in ASTM C273/C273M-16. The effective shear modulus was calculated from the initial linear portion of the numerically obtained load–displacement response as

(1) $$G_{c}=\frac{(\Delta P/\Delta u)t}{Lb}$$

where Δ*P*/Δ*u* is the slope of the initial linear portion of the load–displacement curve, *t* is the core thickness, and *L* and *b* are the length and width of the shear specimen, respectively. The structural mass was directly obtained from the parameterized compression-buckling finite element model in ANSYS. The corresponding material properties, including density, were assigned to the composite face sheets, PET foam core, and PMMA resin-filled regions, and the total structural mass was then extracted directly from the finite element model.

Accordingly, a multi-objective optimization framework is established, aimed at maximizing the critical buckling load, maximizing the shear modulus, and minimizing the structural mass.

The vector of design variables can be formulated as follows:

(2) $$x={\left[w,d,s\right]}^{T}$$

The multi-objective optimization problem is mathematically defined as follows:

(3) $$\left\{\begin{array}{ll} \max & P_{\mathrm{cr}}(x)\\ \max & G_{c}(x)\\ \min & m(x) \end{array}\right.$$

**Figure 10:**
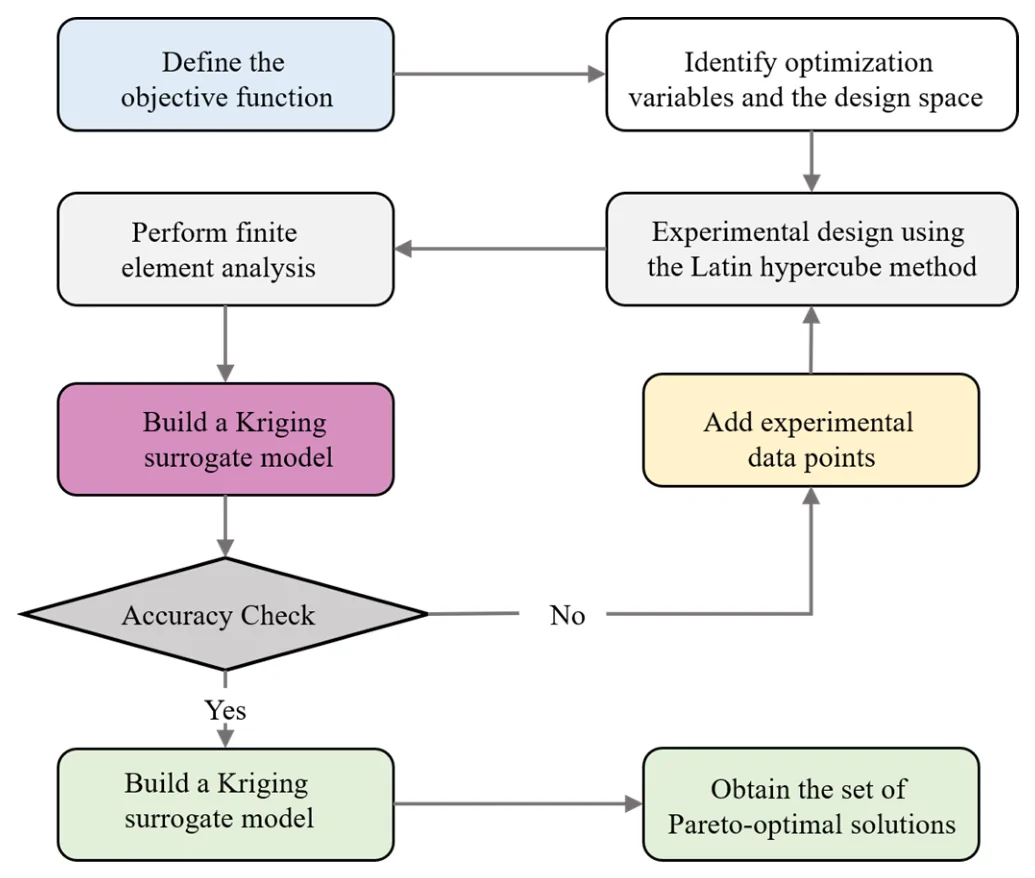
Flowchart of the multi-objective optimization for the grooved PMMA/PET composite foam.

The constraint conditions are defined as follows:

(4) $$\left\{\begin{array}{l} 0.8\;\mathrm{mm}\leq w\leq 2.0\mathrm{mm}\\ 1.5\;\mathrm{mm}\leq d\leq 4.0\mathrm{mm}\\ 15.0\;\mathrm{mm}\leq s\leq 35.0\mathrm{mm} \end{array}\right.$$

where *w*, *d*, and *s* denote the foam groove width, groove depth, and groove spacing, respectively; *P*_cr_ represents the critical buckling load of the sandwich panel; *G*_c_ is the shear modulus; and *m* signifies the structural mass. The bounding ranges of the design variables are synergistically determined by the physical manufacturing feasibility of the specimens, the predefined baseline parametric design boundaries, and the structural liquid infusion molding requirements. The complete flowchart of the above-mentioned multi-objective optimization procedure is illustrated in Figure 10.

During the optimization, the perforation radius was kept constant at *R* = 1 mm, while only the groove width *w*, groove depth *d*, and groove spacing *s* were varied. The foam core adopted a bidirectional orthogonal grooving configuration with identical spacing in both directions.

### 4.2. Construction and Accuracy Verification of the Kriging Metamodel

Multi-objective optimization problems typically necessitate a vast number of parametric combination evaluations. If a finite element model is directly employed to perform the global optimization search, it will inevitably incur prohibitive computational costs, particularly when the design variables vary continuously and the objective functions are nonlinearly coupled; direct optimization yields severely deflated efficiency. To circumvent this limitation, the Latin hypercube sampling (LHS) method was first leveraged in this study to generate 50 sample points within the designated design space. Based on the parametric finite element models constructed in ANSYS, the critical buckling loads, shear moduli, and structural masses under distinct grooving configurations were computed, respectively. Subsequently, taking these finite element results as training datasets, a Kriging surrogate model was established to accurately characterize the highly nonlinear mapping relationships between the foam grooving geometric parameters and the resulting structural responses.

Owing to the pronounced disparities in numerical magnitudes among the critical buckling load, shear modulus, and structural mass, directly applying the raw response values to multi-objective optimization can easily cause indicators with larger magnitudes to dominate the fitness evaluation, thereby diminishing the contributions of other objective functions. To eliminate the interference stemming from disparate dimensions and disparate orders of magnitude on the optimization outcomes, the min–max normalization method was adopted in this study to map each objective response linearly, uniformly shifting them into the [0, 1] interval. The normalization formula is articulated as follows:

(5) $$y_{i}^{*}=\frac{y_{i}-y_{\min}}{y_{\max}-y_{\min}}$$

where *y_i_* represents the raw response value of the *i* sample point; *y*_min_ and *y*_max_ denote the minimum and maximum values of the corresponding response within the entire sample space, respectively; and $y_{i}^{*}$ signifies the normalized response value.

After acquiring the Pareto-optimal solution set, to facilitate engineering evaluation, it is imperative to denormalize the normalized results back into their actual physical quantities. The denormalization formula is articulated as follows:

(6) $$y_{i}=y_{i}^{*}\left(y_{\max}-y_{\min}\right)+y_{\min}$$

Following the construction of the Kriging surrogate model, an additional set of 15 validation sample points was selected to examine the prognostic accuracy of the model. The coefficient of determination (*R*^2^) was adopted as the evaluation metric for the fitting accuracy of the surrogate model, and its mathematical expression is formulated as follows:

(7) $$R^{2}=1-\frac{\sum_{i=1}^{n}{\left(y_{i}-{\hat{y}}_{i}\right)}^{2}}{\sum_{i=1}^{n}{\left(y_{i}-\bar{y}\right)}^{2}}$$

where *y_i_* represents the computed finite element value; ${\hat{y}}_{i}$ is the prognostic value predicted by the Kriging surrogate model; $\bar{y}$ denotes the mean value of the finite element computational results; and *n* is the number of validation samples. An *R*^2^ value closer to 1 indicates a higher consistency between the surrogate model predictions and the finite element computational benchmarks. It is generally accepted that when *R*^2^ > 0.9, the formulated Kriging surrogate model is deemed highly accurate and reliable for subsequent analysis.

**Figure 11:**
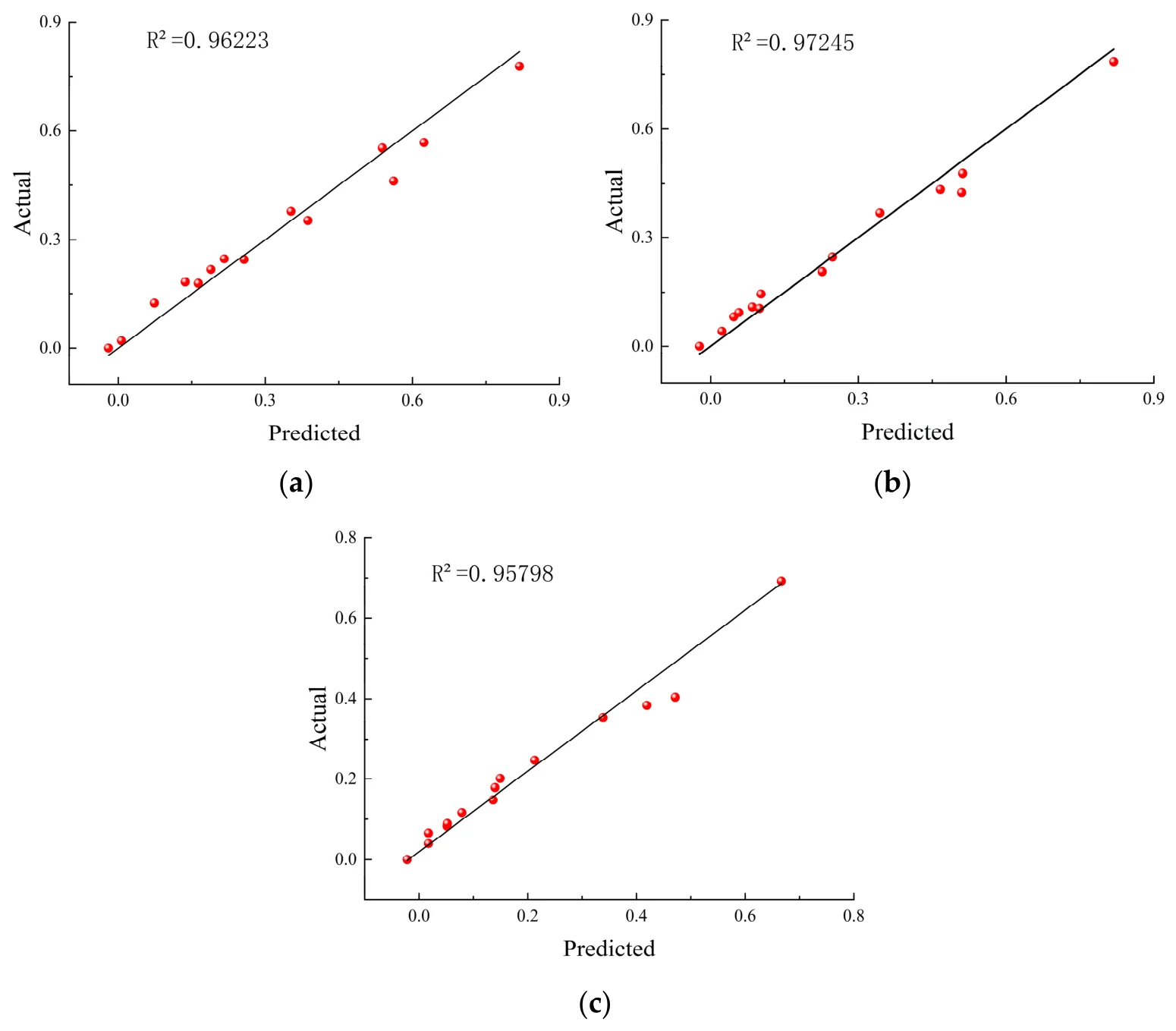
Fitting accuracy of the Kriging surrogate models: (**a**) critical buckling load *P*_cr_; (**b**) foam shear modulus *G*_c_; (**c**) mass *m*. The red dots represent the 15 validation sample points, and the black solid line represents the ideal agreement line (*y* = *x*) between the Kriging-predicted values and the finite element results.

As evidenced by the profiles in Figure 11 and Table 7, the predicted indices for the three distinctive response targets—namely the critical buckling load, shear modulus, and structural mass—are all favorably clustered around their finite element validation points. This high degree of convergence demonstrates that the Kriging framework is capable of accurately characterizing the complex nonlinear relationships that link the grooving geometric parameters to the mechanical performance of the sandwich plates. Quantitatively, the coefficients of determination (*R*^2^) corresponding to the three objective functions are 0.96223, 0.97245, and 0.95798, respectively. The fact that all these statistical metrics consistently exceed the prescriptive 0.9 benchmark firmly underscores the superior fitting precision of the established surrogate model.

### 4.3. Multi-Objective Optimization Results and Analysis Based on NSGA-II

After verifying that the Kriging surrogate model meets the required precision criteria, the non-dominated sorting genetic algorithm II (NSGA-II) was adopted for multi-objective optimization of sandwich panels featuring grooved PMMA/PET composite foams. The main NSGA-II parameters were configured with commonly used values: population size of 30, maximum iterations of 300, crossover probability of 0.8, mutation probability of 0.1, and distribution indices of *η*_c_ = 20 and *η*_m_ = 20 for SBX crossover and polynomial mutation. The optimization objectives are designated to simultaneously maximize the critical buckling load (*P*_cr_), maximize the foam shear modulus (*G*_c_), and minimize the structural mass (*m*). Owing to the inherent trade-offs and mutually constraining relationships among these three physical metrics, no unique absolute optimal solution exists for this multi-objective optimization paradigm; instead, a comprehensive set of Pareto non-dominated solutions is successfully generated.

**Figure 12:**
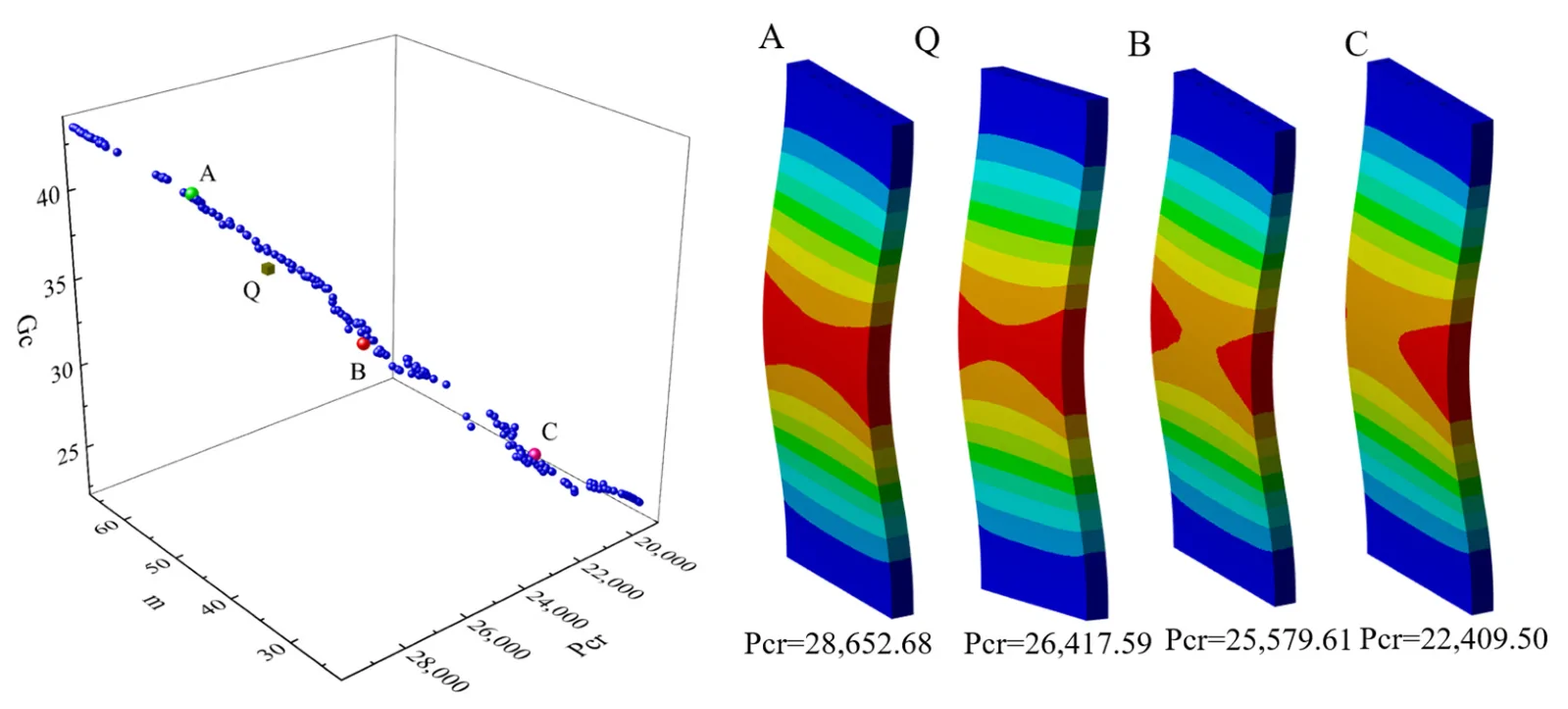
Pareto front solution set and the typical buckling mode shapes corresponding to the key design points. The blue dots represent the Pareto non-dominated solutions. Points A, B, and C represent the recommended, compromise, and lightweight-prioritized designs, respectively, while Point Q denotes the baseline design before optimization. The corresponding buckling mode shapes of Points A, Q, B, and C are shown on the right.

Figure 12 graphically delineates the Pareto-optimal frontier generated via the NSGA-II routine. Herein, labels A, B, and C identify the representative non-dominated configurations along the trade-off boundary, whereas point Q maps the pristine, baseline design prior to optimization.

As observed from Figure 12, the Pareto solution set exhibits a conspicuously continuous distribution, demonstrating that the grooving parameters exert a coupled influence on the buckling load-carrying capacity, shear rigidity, and structural mass of the sandwich plates. Shifting along the Pareto-optimal frontier reveals that an increment in the critical buckling load and foam shear modulus is inevitably accompanied by an inflation in structural mass, whereas a reduction in mass precipitously compromises the mechanical performance. Consequently, in practical engineering scenarios, it is imperative to execute prudent trade-offs among the disparate objectives based on the specific structural service requirements.

To further analyze the performance discrepancies among different optimized solutions, three representative designs denoted as points A, B, and C were selected for comparative cross-examination, with the quantitative results summarized in Table 8.

To establish an explicit engineering criterion for selecting the recommended design, the Pareto solutions were first screened according to a mass-preservation requirement. Considering that an increase in blade mass increases the gravitational, centrifugal, and inertial loads during rotational and flapwise motion, thereby increasing the overall structural load demand, only solutions whose Kriging-predicted structural mass differed from that of the baseline Point Q by no more than 0.10% were considered. Within this mass-constrained subset, Point A exhibited the highest critical buckling load and shear modulus. The Kriging-predicted structural mass of Point A was 52.23, compared with 52.18 for Point Q, corresponding to a difference of approximately 0.096%. Therefore, Point A was selected as the recommended design. Points B and C were retained as representative compromise- and lightweight-prioritized solutions, respectively, to illustrate the trade-offs associated with different design preferences. In practical engineering applications, designers may select an appropriate Pareto solution according to specific service conditions and design requirements.

To rigorously verify the reliability of the optimized outcome, a high-fidelity finite element model was reconstructed utilizing the geometric parameters of point A, and its numerical simulation results were benchmarked against the Kriging model predictions, as summarized in Table 9. The explicit parameters corresponding to point A are determined as: *d* = 3.84 mm, *s* = 16.12 mm, and *w* = 1.37 mm.

As indicated by Table 9, the relative errors for all three structural responses are strictly confined within 5%, thereby demonstrating that the formulated surrogate model possesses adequate fidelity for solving this specific multi-objective optimization problem.

As shown in Table 10, based on the FE results for both configurations, the optimized Point A exhibited a 5.71% increase in critical buckling load and a 10.27% increase in shear modulus compared with the baseline Point Q, while the structural mass decreased by 2.24%. These results confirm that the optimized groove configuration improves both the buckling resistance and shear performance without imposing a mass penalty. These outcomes demonstrate that the judicious configuration of groove width, groove depth, and groove spacing can effectively improve the load-carrying capacity and shear performance of the PMMA/PET grooved sandwich foams. In practical engineering applications, designers may select an appropriate solution from the Pareto set according to specific service conditions and design requirements.

## 5. Conclusions

To address the recycling challenge of thermoset epoxy wind turbine blades, in this study, in-plane compressive buckling tests, finite element (FE) analysis, and multi-objective optimization of the grooving configurations were systematically conducted on the PMMA thermoplastic sandwich panels. The primary conclusions are summarized as follows:

(1) Both PMMA and Epoxy sandwich panels exhibit prominent buckling instability characteristics under in-plane compressive loading, with the ultimate failure mode predominantly governed by global buckling failure. During the loading history, no discernible cracking or fracture is observed in the composite skins. The final structural failure is primarily concentrated in the crushing and cracking of the PET foam, demonstrating that localized foam instability and crushing are the primary mechanisms driving the degradation of load-carrying capacity in the post-buckling regime for such sandwich web architectures.
(2) Compared with the Epoxy/SCP counterparts, the PMMA/SCP sandwich panels exhibit higher buckling load-carrying capacity and a more stable post-buckling response. The average buckling initiation load of the PMMA/SCP sandwich panels is 9.06 kN, representing a 22.1% increase over the Epoxy/SCP specimens, while the average ultimate load reaches 17.93 kN, corresponding to an increase of 5.22%.
(3) By using ANSYS software, a three-dimensional (3D) FE model was established to predict the buckling response of the sandwich panels. The multi-objective optimization results based on the Kriging metamodel and the NSGA-II algorithm demonstrate a distinct coupled influence of the foam grooving parameters on the critical buckling load (*P*_cr_), foam shear modulus (*G*_c_), and structural mass (*m*). The coefficients of determination (*R*^2^) for the established metamodels all exceed 0.95, thereby satisfying the accuracy requirements for optimization calculations. The recommended optimal configuration (Design A) corresponds to the geometric parameters *d* = 3.84 mm, *s* = 16.12 mm, and *w* = 1.37 mm. Compared with the baseline design, the FE-verified recommended configuration reduced the structural mass by 2.24%, while increasing the critical buckling load and foam shear modulus by 5.71% and 10.27%, respectively.

Taking PMMA sandwich panels for wind turbine blade webs and airfoils as the research object, this paper explores their buckling performance and grooved foam designs, offering valuable data references for scaling up the application of eco-friendly PMMA composites in large wind turbine blades.

Future work will incorporate material nonlinearity and progressive damage into the present geometrically nonlinear model, including composite face-sheet failure, foam crushing, and interfacial degradation. Scanning electron microscopy observations of the damaged regions and fracture surfaces will also be conducted to identify and calibrate the governing microscopic failure mechanisms, thereby improving the prediction of post-peak load degradation. In addition, the optimized groove configuration will be fabricated and experimentally tested in future work to further validate the optimization results.

## Figures and Tables

**Table 1:** Mechanical Properties of PMMA and Epoxy Resin.

Material Properties	PMMA	Epoxy
*ρ* (kg/m^3^)	1190	1160
*E* (GPa)	3.372	3.258
*V*	0.37	0.35
*G* (GPa)	1.230	1.207
Tensile strength (MPA)	76.84	72.39

**Table 2:** Properties of T80 PET foam.

Material Properties	Property Value
*ρ* (kg/m^3^)	80
*E* (GPa)	0.065
*V*	0.3
*G* (GPa)	0.054

Note: The properties of PET foam are provided by Beijing Zhongnuo Composite Materials Co., Ltd.

**Table 3:** Properties of PMMA/GF and Epoxy/GF.

Material Properties	PMMA/GF	Epoxy/GF
*E*_1_ (GPa)	36.320	31.100
*E*_2_ (GPa)	6.140	5.374
*V*_12_	0.28	0.30
*V*_23_	0.32	0.35
*G*_12_ (GPa)	2.450	2.100
*ρ* (kg/m^3^)	1903	1897
*V*_f_ (%)	52.8	53.4

**Table 4:** Dimensions and Spacing of Machining Configurations for the foam.

Parameter	*s*/mm	*w*/mm	*d*/mm	*R*/mm
Value	16 × 16	2	3	1

Note: “*s*,” “*w*,” “*d*,” and ‘*R*’ denote slot spacing, slot width, slot depth, and perforation radius, respectively; “*s* = 16 × 16 mm” indicates that both the horizontal and vertical slot spacings are 16 mm.

**Table 5:** Mesh-convergence analysis of the critical buckling load.

Mesh Size (mm)	Number of Elements	Critical Buckling Load (KN)	Relative Error (%)
5.0	24,672	24.266	32.60
4.0	58,868	21.96	20.00
3.0	74,902	20.20	10.38
2.0	130,969	19.07	4.21
1.0	480,257	18.40	0.55
0.8	938,002	18.32	0.11
0.7	1,400,166	18.30	0.00

**Table 6:** Experimental results of compressive buckling for PMMA/SCP and Epoxy/SCP sandwich panels.

Configuration	Specimen	Buckling Initiation Load (kN)	Ultimate Load (kN)	Critical Buckling Load (kN)	Failure Mode
PMMA/SCP	PMMA/Co-a	9.25	17.95	18.40	Global buckling
	PMMA/Co-b	8.99	18.02		Global buckling
	PMMA/Co-c	8.94	17.82		Global buckling
	Average	9.06 ± 0.17	17.93 ± 0.10	—	—
Epoxy/SCP	Epoxy/Co-a	7.62	16.91	17.77	Global buckling
	Epoxy/Co-b	7.46	17.22		Global buckling
	Epoxy/Co-c	7.08	16.99		Global buckling
	Average	7.42 ± 0.28	17.04 ± 0.16	—	—

**Table 7:** Accuracy metrics of the Kriging surrogate models at the 15 validation points.

Objective Function	Critical Buckling Load *P*_cr_	Shear Modulus *G*_c_	Mass *m*
*R* ^2^	0.96223	0.97245	0.95798
MAE (%)	1.64	2.08	4.28
RMSE (%)	1.97	2.45	4.65
Maximum relative error (%)	4.78	5.87	8.43

**Table 8:** Kriging-predicted responses of representative non-dominated solutions on the Pareto frontier.

Optimal Solution	A	B	C
*d*	3.84	3.79	3.76
*s*	16.12	18.52	32.29
*w*	1.37	0.90	0.92
*P*_cr_	28,652.68	25,579.61	22,409.50
*G*_c_	41.45	33.47	27.44
*m*	52.23	40.48	27.83

**Table 9:** Comparison between Kriging-predicted results and finite element simulation results at point A.

	Critical Buckling Load *P*_cr_	Shear Modulus *G*_c_	Mass *m*
FE result	27,926.59	39.93	51.01
Kriging prediction	28,652.68	41.45	52.23
Error (%)	2.6%	3.8%	2.4%

**Table 10:** Comparison of the finite element results between the baseline Point Q and optimized Point A.

	Critical Buckling Load *P*_cr_	Shear Modulus *G*_c_	Mass *m*
Initial point Q (Before optimization)	26,417.59	36.21	52.18
Optimized point A (After optimization)	27,926.59	39.93	51.01
Percentage change	+5.71%	+10.27%	−2.24%

## Data Availability

The original contributions presented in this study are included in the article. Further inquiries can be directed to the corresponding authors.
